# Body composition as a predictor of chemotherapy-related toxicity in ovarian cancer patients: A systematic review

**DOI:** 10.3389/fonc.2022.1057631

**Published:** 2022-11-02

**Authors:** Stefania Rizzo, Giorgio Raia, Maria Del Grande, Maria Luisa Gasparri, Ilaria Colombo, Lucia Manganaro, Andrea Papadia, Filippo Del Grande

**Affiliations:** ^1^ Istituto di Imaging della Svizzera Italiana (IIMSI), Ente Ospedaliero Cantonale (EOC), Lugano, Switzerland; ^2^ Facoltà di Scienze biomediche, Università della Svizzera Italiana, Lugano, Switzerland; ^3^ Istituto Oncologico della Svizzera Italiana (IOSI), Ente Ospedaliero Cantonale (EOC), Bellinzona, Switzerland; ^4^ Department of Gynecology and Obstetrics, Ente Ospedaliero Cantonale (EOC), Lugano, Switzerland; ^5^ Department of Radiological, Oncological and Pathological Sciences, University of Rome Sapienza, Rome, Italy

**Keywords:** ovarian cancer, chemotherapy, body composition, sarcopenia, toxicity

## Abstract

**Objectives:**

The main objective of this systematic review was to examine the literature evaluating association of image-based body composition with chemotherapy-related toxicity in ovarian cancer patients. A secondary objective was to evaluate the different definitions of sarcopenia across studies.

**Methods:**

This systematic review was conducted according to the PRISMA-DTA statement and the protocol was registered on Prospero. A comprehensive literature search of 3 electronic databases was performed by two authors. For each eligible article, information was collected concerning the clinical setting; basic study data; population characteristics; technical aspects; body composition features; chemotherapy drugs administered; association of body composition values and toxicities. The overall quality of the included studies was critically evaluated.

**Results:**

After the initial retrieval of 812 articles, the systematic review included 6 articles (5/6 studies were retrospective; one was prospective). The number of patients ranged between 69 and 239; mean/median age ranged between 55 and 65 years; the percentage of sarcopenic patients ranged between 25% and 54%. The cut-off values to define sarcopenia and the vertebral levels for evaluation of body composition were different. Five studies included chemotherapy based on carboplatin and paclitaxel, 1 included chemotherapy based on pegylated liposomal doxorubicin. Among the studies including carboplatin and paclitaxel, 3/5 demonstrated an association with toxicity, whereas 2/5 did not. Altogether, 4/6 papers demonstrated an association between the body composition values and the development of chemotherapy-related toxicities.

**Conclusions:**

There is a wide variability of results about the association of body composition and chemotherapy-related toxicity in ovarian cancer patients. Therefore further studies, possibly including a comprehensive assessment of body compartments and where the definition of body composition cut-offs is constant, are warranted to better understand this association.

**Systematic review registration:**

https://www.crd.york.ac.uk/prospero/display_record.php?ID=CRD42022337753, identifier (CRD42022337753).

## Introduction

Ovarian cancer (OC) is the second most frequent cancer among gynecological malignancies, with 19.880 estimated new cases in the US in 2022, and the most lethal, with 12.810 estimated deaths (1). The current standard treatment for OC is primary cytoreductive surgery with complete resection of all macroscopic disease, followed by adjuvant platinum-based chemotherapy with or without the antiangiogenic agent bevacizumab (2, 3). When the patient is considered not operable or the disease is deemed not completely resectable, interval debulking surgery after neoadjuvant chemotherapy (NACT) is usually considered (4). In stage III-IV high grade epithelial ovarian cancer, maintenance treatment with poly-ADP-ribose inhibitors (PARPi) has been also incorporate in first line (5–7).

In both scenarios (primary surgery followed by adjuvant chemotherapy, neoadjuvant chemotherapy followed by interval debulking surgery), chemotherapy is dosed aiming at a balance between optimal efficacy and acceptable toxicity. Indeed, if severe toxicity occurs during chemotherapy, the standard chemotherapeutic regimen might not be administered or the dose and schedule adjusted and this might potentially lead to suboptimal treatment and decreased survival. Factors potentially predisposing to toxicity are age, previous chemotherapy, genetic characteristics, including toxicity-related polymorphisms or BRCA mutational status (8, 9). Many authors have hypothesized that body composition, indicating the amount and distribution of muscle and fat compartments, is one of the factors that may predict interpatient variation in toxicity profiles, accounting for different metabolism of chemotherapeutic drugs (10–13). In fact, there is substantial evidence of the variability in body composition in cancer populations (14–16), as well as emerging evidence suggesting that the size of body composition compartments relate to prognosis in many cancer subtypes, including ovarian (17), lung (18), bladder (19) and pancreatic malignancies (20). As demonstrated by some authors, sarcopenic patients may be prone to get higher doses of chemotherapy agents for a rather small amount of muscle mass and they may therefore encounter higher toxicity (21, 22).

Since cancer patients routinely performs imaging examinations during their clinical management (23–25), imaging-based assessment of body composition might be added to the reading of imaging examinations (26, 27), so offering opportunistic clinical information that currently go unused. For instance, from Computed Tomography (CT) images it is possible to extract the areas of muscles at a pre-defined level, usually referred to as skeletal muscle area (SMA); psoas index (PI), indicating only the area of the psoas muscle; the area of visceral adipose tissue (VAT), indicating the fat within the abdomen outside the solid organs; the area of subcutaneous adipose tissue (SAT); the density of the skeletal muscle, as indirect sign of its adipose infiltration (SMD). Despite different definitions and a wide variability of cut-off values for the definition of sarcopenia, this is a condition that can be found in patients with OC and, although many studies have assessed its association with survival, only few studies have assessed the association with chemotherapy-related toxicity.

Therefore, the main objective of this systematic review was to collect and examine all the available literature evaluating association of image-based body composition with chemotherapy-related toxicity in patients with OC. A secondary objective was to evaluate the different definitions of sarcopenia across studies.

## Methods

This systematic review was conducted according to the PRISMA-DTA (Preferred Reporting Items for Systematic Reviews and Meta-analysis for Diagnostic Test Accuracy) statement (28). The review protocol was registered on Prospero as CRD42022337753.

### Search strategy

Two authors (SR and GR) performed a comprehensive literature search of the electronic databases PubMed, Cochrane and Web of Science to find primary publications evaluating association between body composition measures and chemotherapy-related toxicities in OC. No beginning date limit or language restrictions were used; the literature search was last updated on Aug 17^th^ 2022; and the search was expanded by also screening the references of the retrieved articles for additional potentially eligible studies. The search terms consisted of ((ovarian cancer) OR (ovarian carcinoma)) AND ((sarcopenia) OR (body composition) OR (muscle) OR (fat) OR (adipose tissue)) AND ((complication) OR (complications) OR (chemotherapy-related) OR (adjuvant) OR (neo-adjuvant) OR (toxicity) OR (chemotoxicity) OR (chemo-toxicity)). Articles in which body composition assessment was based on CT were obtained in full for further independent evaluation by two authors (SR and GR). There was no exclusion for any type of toxicity and neither for the type or line of chemotherapy. Studies were excluded if they were case reports, conference abstracts, reviews or short communications because they do not provide sufficient information to assess the methodological quality. Uncertainties were resolved in consensus.

### Data extraction

For each eligible article, information was collected by 3 authors (SR, GR, MDG) concerning the clinical setting (neo-adjuvant, adjuvant, further lines); basic study data (year of publication, country of origin, study design); population characteristics (number of patients, age, BMI, percentage of sarcopenic patients, cut-off values for sarcopenia used); technical aspects (axial level for evaluation of body composition); body composition features evaluated (SMA, SMI, VAT, SAT, SMD, PI, lean body mass (LBM), fat mass (FM)); chemotherapy drugs administered; association of body composition values and toxicities.

### Quality assessment

The overall quality of the included studies was critically evaluated based on the revised “Quality Assessment of Diagnostic Accuracy Studies” tool (QUADAS-2) (29). This tool comprises four domains for evaluation of risk of bias (patient selection, index test, reference standard, and flow and timing) and three domains for applicability concerns (patient selection, index test, reference standard). Each domain was assessed and graphs were constructed appropriately.

## Results

### Literature search

**Figure 1:**
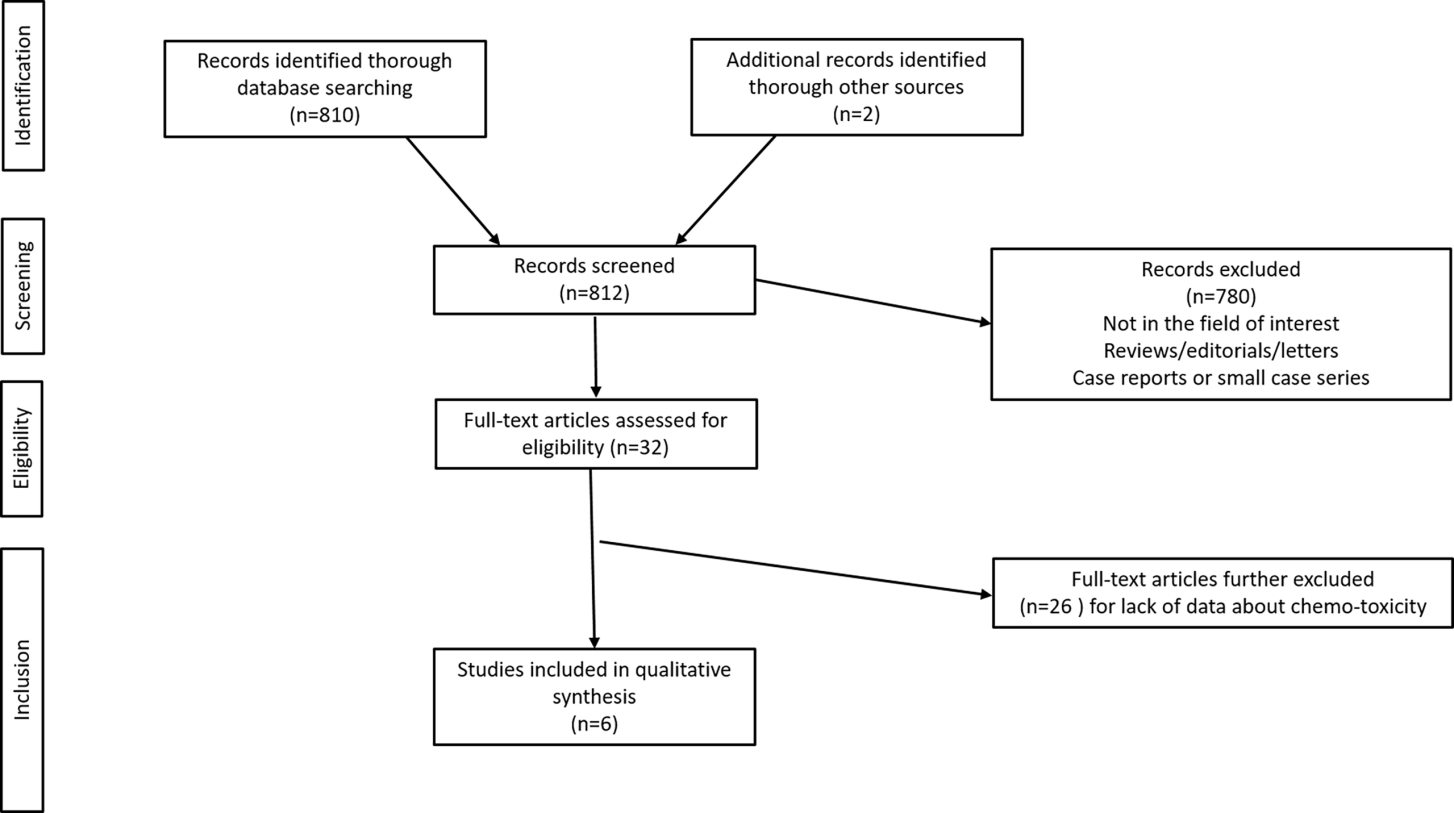
Study selection flowchart.

The initial search yielded 812 articles, all in English. According to inclusion and exclusion criteria, 6 full-text articles were included in this systematic review (17, 30–34). Details about the literature search results are reported in **Figure 1**.

Given the small number of papers included, the clear heterogeneity of the methods and, as a consequence, of the results, it was not possible to perform a meta-analysis for pooled data.

### Basic study data and population characteristics

As shown in **Table 1**, among the 6 studies included, three were from the US (30, 32, 34); the other were from different countries (17, 31, 33). 5/6 studies were retrospective (17, 30–33); one was prospective (from a phase III clinical trial) (34). The number of patients included ranged between 69 (17) and 239 (31); mean/median age ranged between 55 (32, 34) and 65 years (17); the percentage of sarcopenic patients ranged between 25% (17) and 54% (32). The BMI ranged between 24.9 (17) and 28 (32). The cut-off values to define sarcopenia in different studies and the percentage of sarcopenic patients are summarized in **Table 1**.

**Table 1:** Basic study and population characteristics.

Authors	Year	Country	Study design	N patients	Mean/median age (years)	BMI (mean)	Percentage of sarcopenic patients	Cut-off values for sarcopenia
Prado (29)	2014	US	Prospective	74	55	27.9	NA	NA
Yoshikawa (28)	2017	Japan	Retrospective	76	62	NA	50%	PI < 58.3 cm^2^/m^2^
Conrad (27)	2018	US	Retrospective	102	55	28	54%	PI<38.5 cm^2^/m^2^
Staley (25)	2020	US	Retrospective	201	64	26.9	27%	SMI<41 cm^2^/m^2^
Bruno (26)	2021	Brazil	Retrospective	239	56	NA	35%	SMI<38.9 cm^2^/m^2^
Del Grande (12)	2021	Switzerland	Retrospective	69	65	24.9	25%	SMI<41 cm^2^/m^2^

NA, not available; BMI, body mass index; SMI, skeletal muscle index; PI, psoas index.

### Body composition evaluation details; chemotherapy administered and association of body composition to chemo-related toxicity

As shown in **Table 2**, 4/6 articles evaluated the body composition values at the level of the 3rd lumbar vertebra (L3) (17, 30, 31, 34); 1/6 at the level of the 4th lumbar vertebra (L4) (32); 1/6 at the level of the 5th lumbar vertebra (L5) (33). The main body composition parameters evaluated were: SMI (derived from SMA) in 3/6 studies (17, 30, 31); psoas index (PI) in 2/6 studies (32, 33); SAT in 3/6 studies (17, 31, 32); SMD in 3/6 (17, 31, 34). Five studies included chemotherapy based on carboplatin and paclitaxel (17, 30– 33), 1 included chemotherapy based on pegylated liposomal doxorubicina (34).

**Table 2:** Body composition evaluation details; chemotherapy administered and association of body composition to chemo-related toxicity (if any).

Authors	Vertebra level for body composition assessment	Body composition features evaluated	Chemotherapy	Association of body composition and chemo-related toxicity
Prado (29)	L3	SMD; LBM; FM	Pegylated liposomal doxorubicina	FM/LBM ratio associated with toxicity only in overweight and obese patients
Yoshikawa (28)	L5	PI	Carboplatin and paclitaxel	PI associated with neuropathy
Conrad (27)	L4	PA; PI, VAT, SAT	Carboplatin and paclitaxel	No association
Staley (25)	L3	SMA, SMI	Carboplatin and paclitaxel	No association
Bruno (26)	L3	SMI, SAT, SMD	Carboplatin and paclitaxel	SAT and SMD associated with G3 adverse events and toxicity-induced modification of treatment
Del Grande (12)	L3	SMA, SMI, VAT, SAT, SMD	Carboplatin and paclitaxel	VAT and SMD associated with chemotherapy cycle delays; SMA with early discontinuation of chemotherapy

L3, 3^rd^ lumbar vertebra; L4, 4^th^ lumbar vertebra; L5, 5^th^ lumbar vertebra; SMA, skeletal muscle area; SMI, skeletal muscle index; VAT, visceral adipose tissue; SAT, subcutaneous adipose tissue; SMD, skeletal muscle density; PA, psoas area; PI, psoas index; LBM, lean body mass; FM, fat mass; G3, grade 3.

Two studies (12, 28) included both the neo-adjuvant and adjuvant settings; one declared only the first line setting (25); one included only patients treated with a further line treatment (29). Among the studies including carboplatin and paclitaxel, 3/5 demonstrated an association with toxicity (17, 31, 33), whereas 2/5 did not (30, 31). Altogether, 4/6 papers demonstrated an association between the body composition values and the development of chemotherapy-related toxicities (17, 31, 33, 35), with one showing association of VAT and SMD with chemotherapy cycle delays as well as of SMA and early discontinuation of chemotherapy (17); one showing an association of SAT and SMD with G3 adverse events and toxicity-induced modification of treatment (31); one showing association of the psoas index with neuropathy (33); one showing association of the FM/LBM ratio with toxicity only in overweight and obese patients (35).

### Quality assessment of the studies included

**Figure 2:**
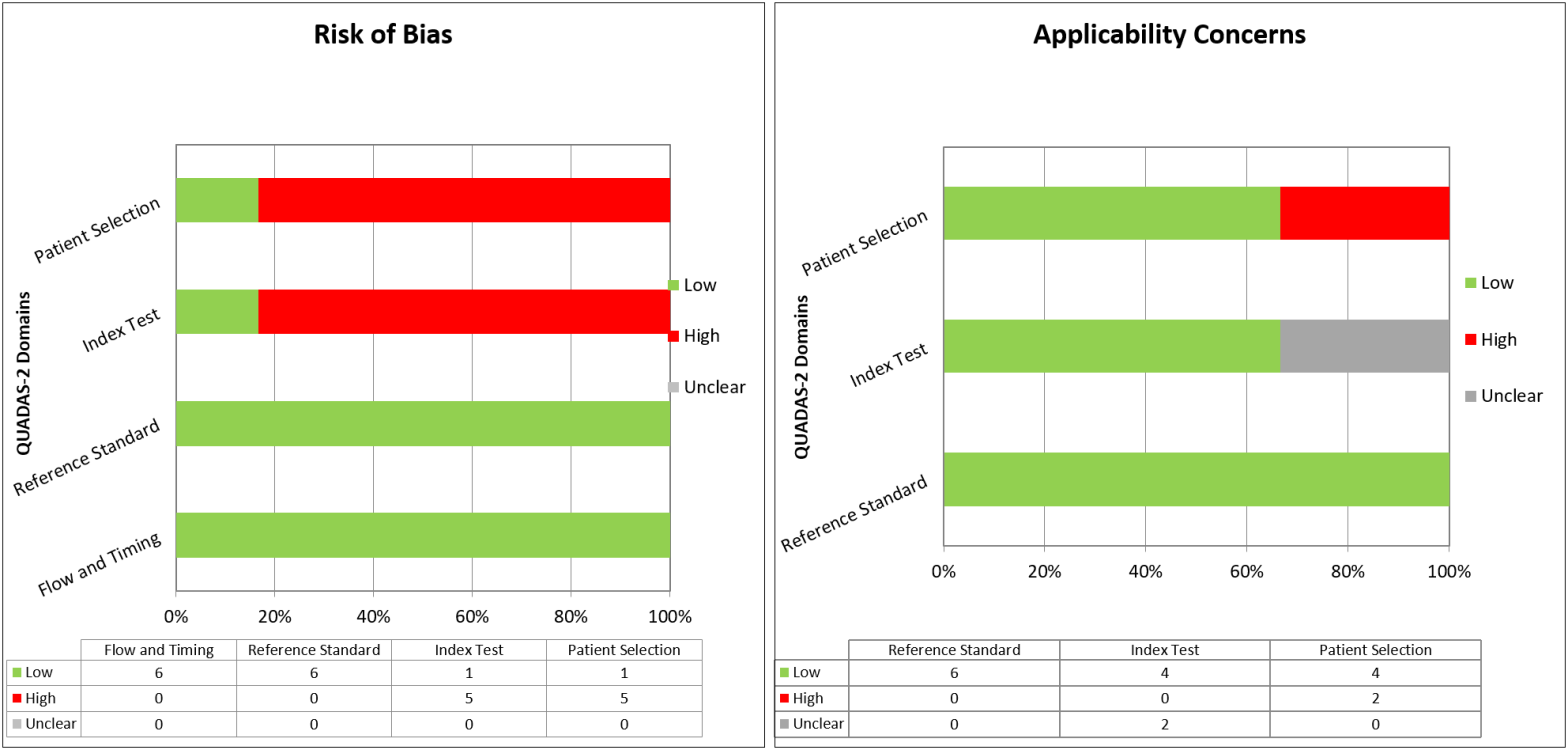
Overall quality assessment (risk of bias and applicability concerns) of the studies included in the Systematic Review, according to the QUADAS-2 Tool.

The overall quality assessment of the studies is reported in **Figure 2**.

## Discussion

This systematic review demonstrates that the association between body composition and chemo-related toxicity in OC is still unclear. Indeed, 4/6 studies demonstrated the presence of a significant association, but 2/6 did not. Furthermore, the significant associations were not among the same covariates across studies.

Interestingly, the 2 studies showing no significant association of body composition and toxicity are from the same country (US) (30, 32), that is also known for its high percentage of overweight/obese patients. Indeed, the age-adjusted prevalence of obesity in the US in 2017–2018 was 42.4%, and the age-adjusted prevalence of severe obesity was as high as 9.2% among adults (>20 years), especially among women (35). This high prevalence of overweight/obese patients, confirmed by the high mean BMI in both studies, might have affected the results. Indeed, the low muscle mass may be underestimated in obese patients.

What emerges from the analysis of the results of the included studies, is that no study evaluated all the body compartments available, therefore some information is still missing. Indeed, for instance, Del Grande showed an association of SMA and early discontinuation of chemotherapy, but SMI was not significant (17). In the same study VAT and SMD were significantly associated with cycle delays, but SAT was not (17). Bruno et al. demonstrated the importance of SAT and SMD for G3 adverse events, but they did not evaluate the other compartments (31). Conrad and Yoshikawa analyzed only the PI, thus excluding all the other muscles in the same plane and even the other body compartments (32, 33).

Nevertheless, various methods are available for assessing body composition and they are based on an escalating level of complexity, from a two-compartment model (evaluating only fat mass and fat free mass), through a three-compartment model (including fat mass, lean tissue mass and bone mineral content), and a four-compartment model (including fat, mineral, total body water and proteins) to more complex multi-compartment models (including complex measurements of elements such as calcium, sodium, chloride, phosphorus, nitrogen, hydrogen, oxygen and carbon) (36). Therefore, other studies hypothesized that not only the quality and quantity of muscle are important in the metabolism of drugs, but also other compartments may contribute to the metabolism of chemotherapeutic agents (37). Indeed, the body proportions of lean and adipose tissues may be one of the phenotypic factors that affect the metabolism, clearance, and toxicity of antineoplastic agents (38). Accordingly, Schachar et al. analyzed a large number of body composition measures to assess predictors of toxicity in patients receiving chemotherapy for early stage breast cancer, and they demonstrated that body composition is extremely variable, demonstrating in their cohort that muscle metrics were clearly related to toxicity, whereas adipose metrics were not (39). Other studies tried to integrate the information of quality and quantity of muscle introducing a relatively new metric, a product of SMI and SMD (40), and demonstrated that this metric predicted G4 hematologic and G3/4 non-hematologic adverse event toxicity when eribulin was administered as a treatment in advanced soft tissue sarcoma (41).

As the technology advances, we may imagine that more comprehensive body composition quantifications will be possible as opportunistic assessments from imaging studies in patients with OC, including but not limited to assessment of bone mineral density, quantification of visceral and subcutaneous fat, assessment of muscle bulk and density, and quantification of liver fat (42).

This systematic review has some limitations. The first is the lack of a prospective cohort study evaluating the association of body composition and chemotherapy-related composition as primary objective. However, this type of study is difficult to obtain and usually have prognosis as primary outcome. Secondly, we included studies where the body composition was based on CT images and we do not know if other studies, based on DEXA or other techniques may show different results. However, CT (along with magnetic resonance) is currently considered as gold standard for assessment of body composition, therefore we may affirm that the data collected are reliable among the included studies. Lastly, the variability of definition of sarcopenia among the included studies, and the lack of reasons for the authors to choose different cut-offs, makes difficult an appropriate comparison. Indeed, we cannot know if the use of the same cut-off value for sarcopenia, would have led to more consisting results.

In conclusion, this systematic review of the literature demonstrated that there is a wide variability of results about the association of body composition and chemotherapy-related toxicity in patients with OC. Therefore further studies, possibly including a comprehensive assessment of body compartments and a constant definition of body composition cut-offs, are warranted to better understand this association.

## Data availability statement

The original contributions presented in the study are included in the article/supplementary material. Further inquiries can be directed to the corresponding author.

## Author contributions

Conception and design: all authors. Data extraction from included studies: SR, GR, MDG, MLG. Analysis and interpretation of data: SR, GR, MDG, MLG. Manuscript writing: all authors. All authors contributed to the article and approved the submitted version.

## Conflict of interest

The reviewer FT declared a shared affiliation with the author LM to the handling editor at the time of the review.

The remaining authors declare that the research was conducted in the absence of any commercial or financial relationships that could be construed as a potential conflict of interest.

## Publisher’s note

All claims expressed in this article are solely those of the authors and do not necessarily represent those of their affiliated organizations, or those of the publisher, the editors and the reviewers. Any product that may be evaluated in this article, or claim that may be made by its manufacturer, is not guaranteed or endorsed by the publisher.
